# Closed-Form Equation of Data Dependent Jitter in First Order Low Pass System

**DOI:** 10.1155/2014/694178

**Published:** 2014-10-16

**Authors:** Sangjin Byun

**Affiliations:** Division of Electronics and Electrical Engineering, Dongguk University-Seoul, 26 Pil-dong 3-ga, Jung-gu, Seoul 100-715, Republic of Korea

## Abstract

This paper presents a closed-form equation of data dependent jitter (DDJ) in first order low pass systems. The DDJ relates to the system bandwidth, the bit rate, the input rise/fall time, and the number of maximum consecutive identical bits of the data pattern. To confirm the derived equation, simulations have been done with a first order RC low pass circuit for various system bandwidths, bit rates, input rise/fall times, and data patterns. The simulation results agree well with the calculated DDJ values by the derived equation.

## 1. Introduction

As bit rate increases, timing jitter becomes more critical to system performances of a high speed serial interface. Timing jitter deteriorates signal quality at the transmitter side and degrades BER performance at the receiver side [1–4]. To guarantee the satisfactory system performances of a high speed serial interface, timing jitter should be accurately predicted and carefully considered when we design system architecture, link budget, and each circuit building block.

**Figure 1:**
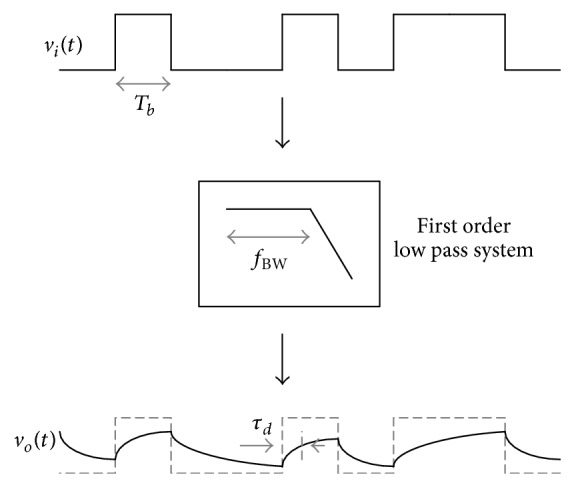
Response of first order low pass system.

Timing jitter is composed of unbounded random jitter (RJ) and bounded deterministic jitter (DJ). The RJ is produced by Gaussian electrical noise within system components and the DJ is categorized into duty cycle distortion (DCD) jitter, data dependent jitter (DDJ), and bounded uncorrelated jitter (BUJ) [5, 6]. Among them, the DDJ is focused on in this paper. The DDJ has an impact on the high speed serial interface especially when the bit rate increases while the system bandwidth is restricted [7, 8]. As shown in Figure 1, the DDJ is generated when a certain data pattern with the bit rate, *T*_*b*_, passes through a system with the limited bandwidth, *f*_BW_.

So far, some papers have been published to predict the DDJ in the general transmission lines [7–9] and in the first order low pass systems [10–14]. The DDJ in the transmission line may apply to the interconnect channels such as off-chip PCB traces, off-chip cables, and on-chip interconnect lines while the DDJ in the first order low pass system may apply to the transceiver circuit building blocks such as drivers, buffers, amplifiers, and limiters. For the transmission lines, the DDJ has been predicted by using the simulated transient step response and the worst-case input bit sequence to shorten the simulation time [7–9]. On the other hand, for the first order low pass systems, the DDJ has been predicted based on the infinite number of calculated pulse or step responses of all the previous bits while the rise/fall time of the input signal was assumed to be zero ideally. However, because it is not possible to calculate the infinite number of pulse or step responses of all the previous bits, only two or four preceding bits have been considered instead for the actual DDJ prediction [10–14]. So, the calculated DDJ always underestimates the real DDJ and the prediction accuracy may degrade as the bit rate increases relatively to the system bandwidth.

In this paper, a new closed-form equation of DDJ in the first order low pass system is presented. The DDJ is directly derived by solving the differential equation of the first order RC low pass circuit and by using the repetitiveness of the data pattern. Of course, this repetitiveness of the data pattern can be generalized for the real random data by increasing the pattern length to the infinity. The derived DDJ equation relates to the system bandwidth, the bit rate, the input rise/fall time, and the number of maximum consecutive identical bits of the data pattern. Contrary to the previous works, the calculated DDJ by the derived equation coincides exactly with the simulated DDJ. Additionally, the effect of nonzero input rise/fall time is also to be considered.

This paper is composed of five sections. In Section 2, the closed-form DDJ equation is derived by assuming zero input rise/fall time.  Section 3 extends the derived DDJ equation to the data pattern with nonzero input rise/fall time. To confirm the derived DDJ equation, the simulation results are shown in Section 4 and conclusions are given in Section 5.

## 2. Calculation of DDJ with Zero Input Rise/Fall Time

### 2.1. Differential Equation of First Order RC Low Pass Circuit

**Figure 2:**
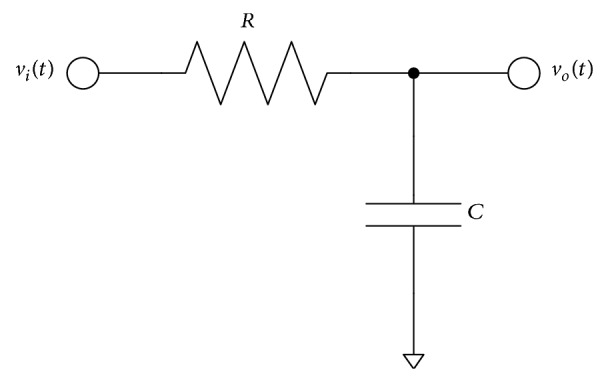
First order RC low pass circuit.

**Figure 3:**
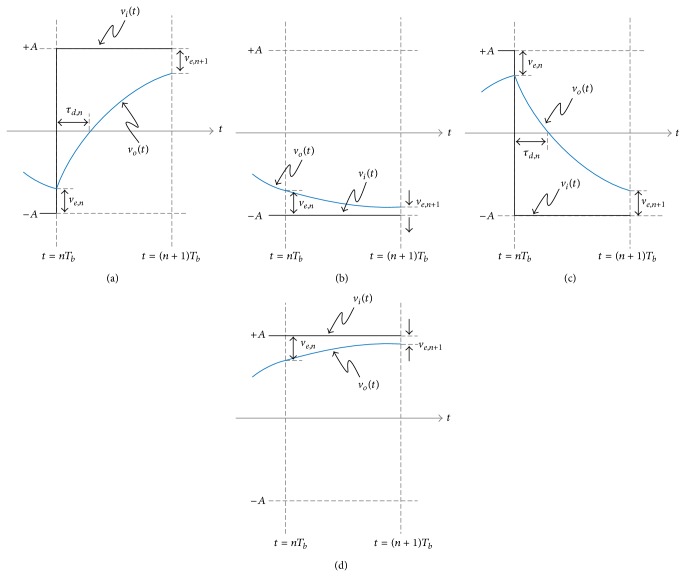
Input and output waveforms when the bit transition pattern is (a) 01, (b) 00, (c) 10, and (d) 11 and the input rise/fall time is zero.

Given a data pattern, a bit rate, and a system bandwidth, the DDJ can be derived by solving the differential equation of the first order RC low pass circuit shown in Figure 2. Depending on the bit transition patterns, such as 01, 00, 10, and 11, the input signal, *v*_*i*_(*t*), and the initial condition, *v*_*o*_(*nT*_*b*_), of the first order RC low pass circuit are given differently for each bit duration, *T*_*b*_, as shown in Figure 3. In the figure, the input rise/fall time is zero ideally. So, *v*_*i*_(*t*) is −*A* or +*A* for *nT*_*b*_ < *t* ≤ (*n* + 1)*T*_*b*_ and a new variable, *v*_*e*,*n*_, is defined as the voltage difference between *v*_*i*_(*t*) and *v*_*o*_(*t*) at *t* = *nT*_*b*_, where *n* is an integer; that is, *v*_*e*,*n*_ = |*v*_*i*_(*nT*_*b*_) − *v*_*o*_(*nT*_*b*_)|. Also, for the bit transition pattern of 01 or 10, another variable, *τ*_*d*,*n*_, is defined as the time difference between the threshold crossing times of *v*_*i*_(*t*) and *v*_*o*_(*t*), as shown in Figures 3(a) and 3(c). The definition of the variables can be found in Table 1.

Because there are four different sets of the input signal, *v*_*i*_(*t*), and the initial condition, *v*_*o*_(*nT*_*b*_), depending on the bit transition patterns, the below differential equation of the first order RC low pass circuit should be solved for each bit transition pattern:

(1) $$\begin{array}{c} \frac{v_{o}\left(t\right)-v_{i}\left(t\right)}{R}+C\frac{dv_{o}\left(t\right)}{dt}=0. \end{array}$$

First, when the bit transition pattern is 01 as shown in Figure 3(a), the initial condition and the input signal are *v*_*o*_(*nT*_*b*_) = −*A* + *v*_*e*,*n*_ and *v*_*i*_(*t*) = *A* for *nT*_*b*_ < *t* ≤ (*n* + 1)*T*_*b*_, respectively. Then, the output signal is

(2) vo(t)=vo(nTb)+{A−vo(nTb)}(1−e−(t−nTb)/RC)for  nTb≤t≤(n+1)Tb.

By using that *v*_*o*_((*n* + 1)*T*_*b*_) = *A* − *v*_*e*,*n*+1_, the relationship between *v*_*e*,*n*_ and *v*_*e*,*n*+1_ is obtained as

(3) $$\begin{array}{c} v_{e,n+1}=2Ar-v_{e,n}r, \end{array}$$

where *r* = *e* ^−(*T*_*b*_/RC)^ and, by using that *v*_*o*_(*nT*_*b*_ + *τ*_*d*,*n*_) = 0, *τ*_*d*,*n*_ is obtained as a function of *v*_*e*,*n*_:

(4) $$\begin{array}{c} \tau_{d,n}=T_{b}\;\text{lo}{\text{g}}_{r}\left(\frac{A}{2A-v_{e,n}}\right). \end{array}$$

Second, when the bit transition pattern is 00 as shown in Figure 3(b), *v*_*o*_(*nT*_*b*_) = −*A* + *v*_*e*,*n*_ and *v*_*i*_(*t*) = −*A* for *nT*_*b*_ < *t* ≤ (*n* + 1)*T*_*b*_. Then, the output signal is

(5) vo(t)=vo(nTb)+{−A−vo(nTb)}(1−e−(t−nTb)/RC)for  nTb≤t≤(n+1)Tb

and, by using that *v*_*o*_((*n* + 1)*T*_*b*_) = −*A* + *v*_*e*,*n*+1_, another relationship between *v*_*e*,*n*_ and *v*_*e*,*n*+1_ is obtained:

(6) $$\begin{array}{c} v_{e,n+1}=v_{e,n}r. \end{array}$$

Finally, when the bit transition pattern is 10 or 11 as shown in Figures 3(c) or 3(d), the same relationship between *v*_*e*,*n*_ and *v*_*e*,*n*+1_ can be obtained as (3) or (6) and *τ*_*d*,*n*_ can be obtained as (4) because Figures 3(c) and 3(d) are just vertically symmetric with Figures 3(a) and 3(b). In summary, (3) and (6) describe how *v*_*e*,*n*_ is updated to *v*_*e*,*n*+1_ per every *T*_*b*_ according to the bit transition pattern and (4) describes how *τ*_*d*,*n*_ relates to *v*_*e*,*n*_ when a bit transition occurs.

### 2.2. DDJ Calculation

By using (3), (6), and (4), the DDJ can be derived through the following steps.

**Figure 4:**
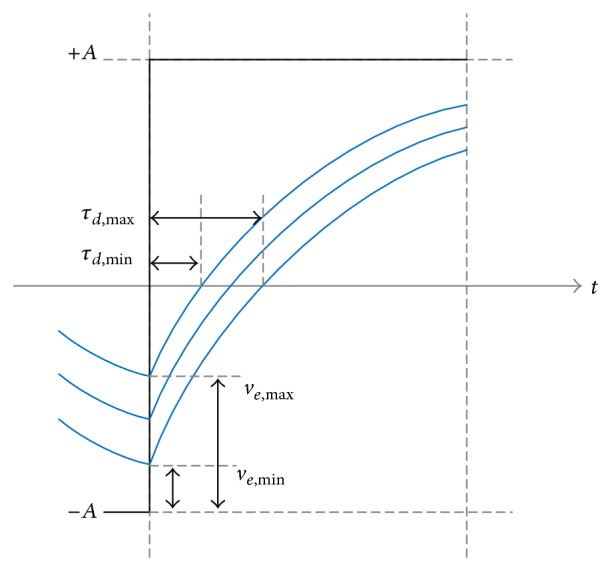
Relationship between *v*_*e*,*n*_ and *τ*_*d*,*n*_.

- Calculate *v*_*e*,*n*_ by using (3) and (6) for the repeated data pattern with the finite pattern length of *N*.
- Find the maximum and minimum values, *v*_*e*,max⁡_ and *v*_*e*,min⁡_, among the set of the calculated *v*_*e*,*n*_ values.
- Calculate *τ*_*d*,min⁡_ and *τ*_*d*,max⁡_ corresponding to *v*_*e*,max⁡_ and *v*_*e*,min⁡_ by using (4). Note that *τ*_*d*,*n*_ is inversely proportional to *v*_*e*,*n*_ as shown in Figure 4.
- Finally, DDJ = *τ*_*d*,max⁡_ − *τ*_*d*,min⁡_. For random data with the infinite pattern length, the DDJ equation should be modified appropriately.

**Figure 5:**
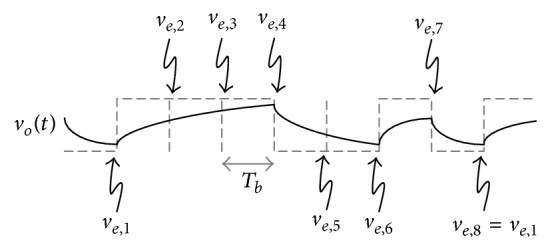
Output waveform when the input data pattern is PRBS3 as an example.

If a data pattern has the finite pattern length of *N* and passes through a first order RC low pass circuit in a steady state, there should exist *N* different values of *v*_*e*,*n*_ in the output waveform. For example, if a data pattern is PRBS3, the pattern length is 7 and *v*_*e*,*n*_ is always mapped to one of {*v*_*e*,1_, *v*_*e*,2_, *v*_*e*,3_, *v*_*e*,4_, *v*_*e*,5_, *v*_*e*,6_, *v*_*e*,7_} as shown in Figure 5. However, all of *v*_*e*,*n*_ do not need to be considered for calculation of the DDJ. Among {*v*_*e*,1_, *v*_*e*,2_, *v*_*e*,3_, *v*_*e*,4_, *v*_*e*,5_, *v*_*e*,6_, *v*_*e*,7_}, *v*_*e*,1_, *v*_*e*,4_, *v*_*e*,6_, and *v*_*e*,7_ are needed because only they are at the bit transition edges of PRBS3. Thus, if the number of bit transitions within a data pattern is *K*, only *K* values of *v*_*e*,*n*_ need to be considered for calculation of the DDJ. On the other hand, *v*_*e*,*n*_ has Markov property [15, 16]. A variable is said to have Markov property if the future value depends only on the present value and not on the past values. As seen from (3) and (6), *v*_*e*,*n*+1_ depends only on *v*_*e*,*n*_ and not on the preceding values of *v*_*e*,*n*_ such as *v*_*e*,*n*−1_ and *v*_*e*,*n*−2_. Thus,

(7) ve,2=2Ar−ve,1r,  ve,3=ve,2r,ve,4=ve,3r,  ve,5=2Ar−ve,4r,ve,6=ve,5r,  ve,7=2Ar−ve,6r,ve,8=2Ar−ve,7r.

By using the repetitiveness of PRBS3, *v*_*e*,8_ = *v*_*e*,1_ and, thus, *v*_*e*,1_, *v*_*e*,4_, *v*_*e*,6_, and *v*_*e*,7_ are obtained as follows:

(8) ve,1=2Ar1−r+r3−r61−r7

(9) ve,4=2Arr2−r3+r4−r61−r7

(10) ve,6=2Arr−r4+r5−r61−r7

(11) ve,7=2Ar1−r2+r5−r61−r7.

**Figure 6:**
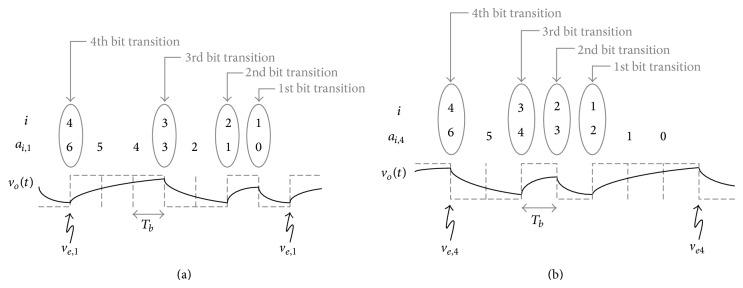
How to determine (a) *a*_*i*,1_ of *v*_*e*,1_ and (b) *a*_*i*,4_ of *v*_*e*,4_ when the input data pattern is PRBS3 as an example.

Before finding *v*_*e*,max⁡_ and *v*_*e*,min⁡_ from (8) to (11), *v*_*e*,*n*_ can be generalized to

(12) $$\begin{array}{c} v_{e,n}=2Ar\frac{\sum_{i=1}^{K}{\left(-1\right)}^{i+1}r^{a_{i,n}}}{1-r^{N}} \end{array}$$

for any data pattern with the finite pattern length of *N*. Here, *K* is the number of bit transitions and *a*_*i*,*n*_ is an integer variable defined as the relative bit distance of the *i*th bit transition backwards from *v*_*e*,*n*_, where *a*_*i*,*n*_ ∈ {0,1,…, *N* − 1}. In (12), *a*_*i*,*n*_ is determined by the relative bit transition positions within the data pattern because the relationship between *v*_*e*,*n*+1_ and *v*_*e*,*n*_ is determined by (3) whenever a bit transition occurs like 01 or 10 and by (6) whenever a bit holds like 00 or 11.  Figure 6 shows that *a*_1,1_ = 0, *a*_2,1_ = 1, *a*_3,1_ = 3, and *a*_4,1_ = 6 for *v*_*e*,1_ and *a*_1,4_ = 2, *a*_2,4_ = 3, *a*_3,4_ = 4, and *a*_4,4_ = 6 for *v*_*e*,4_, respectively, as an example. The obtained values of *a*_*i*,*n*_ in Figure 6 agree well with (8) and (9). Thus, *v*_*e*,*n*_ can be generally represented as (12) for any data pattern with the finite pattern length of *N* if the data pattern is known.

Now, *v*_*e*,max⁡_ and *v*_*e*,min⁡_ can be found among {*v*_*e*,1_, *v*_*e*,2_,…, *v*_*e*,*N*_}. If *v*_*e*,*m*_ = 2*Ar*(∑_*i*=1_^*K*^(−1)^*i*+1^ *r* ^*a*_*i*,*m*_^/(1 − *r* ^*N*^)) and *v*_*e*,*n*_ = 2*Ar*(∑_*i*=1_^*K*^(−1)^*i*+1^ *r* ^*a*_*i*,*n*_^/(1 − *r* ^*N*^)), where *m* ≠ *n*, *v*_*e*,*m*_ and *v*_*e*,*n*_ can be compared by using the following theorems, of which proofs are given in Appendix A.

**Theorem 1 .**

If *a*_1,*m*_ > *a*_1,*n*_, then *v*_*e*,*m*_ < *v*_*e*,*n*_.

**Theorem 2 .**

If *a*_*i*,*m*_ = *a*_*i*,*n*_  for all *i* = 1,…, 2*k* − 1, where *k* is an integer and *a*_2*k*,*m*_ > *a*_2*k*,*n*_, then *v*_*e*,*m*_ > *v*_*e*,*n*_.

**Theorem 3 .**

If *a*_*i*,*m*_ = *a*_*i*,*n*_ for all *i* = 1,…, 2*k*, where *k* is an integer and *a*_2*k*+1,*m*_ > *a*_2*k*+1,*n*_, then *v*_*e*,*m*_ < *v*_*e*,*n*_.

For PRBS3, *v*_*e*,min⁡_ = *v*_*e*,4_ by Theorem 1 because *a*_1,1_ = *a*_1,7_ = 0 < *a*_1,6_ = 1 < *a*_1,4_ = 2 and *v*_*e*,max⁡_ = *v*_*e*,7_ by Theorem 2 because *a*_2,1_ = 1 < *a*_2,7_ = 2 as seen from (8)~(11). These theorems can be generally applied to any data pattern with the finite pattern length. However, if a data pattern is random and has the infinite pattern length, there are infinite numbers of *v*_*e*,*n*_ and so *v*_*e*,max⁡_ and *v*_*e*,min⁡_ are obtained in the different way. In that case, the number of maximum consecutive identical bits is also infinite so that *v*_*e*,min⁡_ = 0 from (12) and *v*_*e*,max⁡_ = 2*Ar* from (3).

After *v*_*e*,max⁡_ and *v*_*e*,min⁡_ are found, *τ*_*d*,max⁡_ and *τ*_*d*,min⁡_ are obtained by (4) as

(13) $$\begin{array}{c} \tau_{d,\max}=T_{b}\;\text{lo}{\text{g}}_{r}\left(\frac{A}{2A-v_{e,\min}}\right)\\ \tau_{d,\min}=T_{b}\;\text{lo}{\text{g}}_{r}\left(\frac{A}{2A-v_{e,\max}}\right) \end{array}$$

and the DDJ is finally derived as

(14) $$\begin{array}{c} \text{DDJ}=\tau_{d,\max}-\tau_{d,\min}=T_{b}\;\text{lo}{\text{g}}_{r}\left(\frac{2A-v_{e,\max}}{2A-v_{e,\min}}\right). \end{array}$$

Thus, for PRBS3,

(15) $$\begin{array}{c} \text{DDJ}=T_{b}\;\text{lo}{\text{g}}_{r}\left(\frac{1-r+r^{3}-r^{6}}{1-r^{3}+r^{4}-r^{5}}\right) \end{array}$$

and, for random data,

(16) $$\begin{array}{c} \text{DDJ}=T_{b}\;\text{lo}{\text{g}}_{r}\left(1-r\right). \end{array}$$

Additionally, for other PRBS data patterns like PRBS4 and PRBS5, the DDJ can be derived as shown in Appendix B. Carefully observing (15), (16), (B.1), and (B.2), the DDJ can be generally approximated to

(17) $$\begin{array}{c} \text{DDJ}\approx T_{b}\;\text{lo}{\text{g}}_{r}\left(\frac{1-r}{1-r^{M}}\right) \end{array}$$

for any data pattern by using the number of maximum consecutive identical bits, *M*. For any PRBS *M* data patterns, the number of maximum consecutive identical bits equals *M*. Finally, the calculated *v*_*e*,max⁡_, *v*_*e*,min⁡_, *τ*_*d*,max⁡_, *τ*_*d*,min⁡_, and DDJ are compared for various data patterns in Table 2. The DDJ of PRBS *M* approaches the DDJ of random data as the number of maximum consecutive identical bits, *M*, increases to the infinity.

## 3. Calculation of DDJ with Nonzero Input Rise/Fall Time

### 3.1. Differential Equation of First Order RC Low Pass Circuit

**Figure 7:**
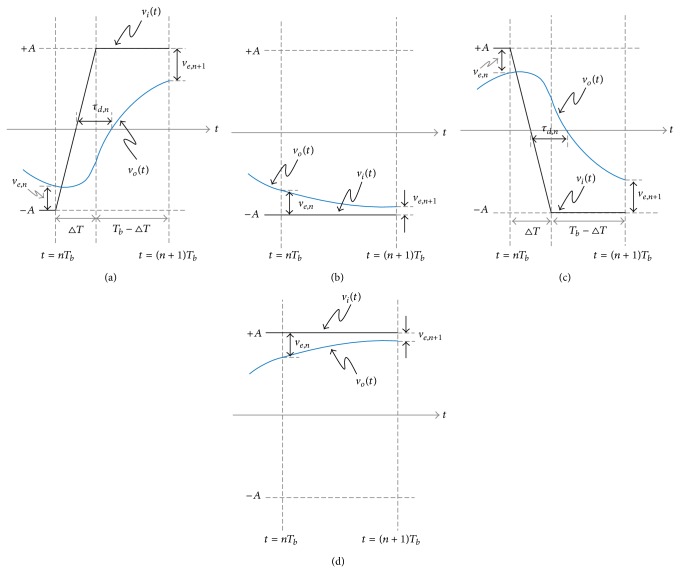
Input and output waveforms when the bit transition pattern is (a) 01, (b) 00, (c) 10, and (d) 11 and the input rise/fall time is nonzero.

Now, the effect of the nonzero input rise/fall time on the DDJ can be considered. The differential equation of (1) should be solved again for four different bit transition patterns, such as 01, 00, 10, and 11, when the input rise/fall time is Δ*T* as shown in Figure 7. Although there are more accurate models for the rising/falling edges of the input signal, *v*_*i*_(*t*), the first order model is adopted for simplicity of calculation to derive the closed-form DDJ equations in this paper.

First, when the bit transition pattern is 01 as shown in Figure 7(a), the initial condition is *v*_*o*_(*nT*_*b*_) = −*A* + *v*_*e*,*n*_ and the input signal is *v*_*i*_(*t*) = −*A* + (2*A*/Δ*T*)(*t* − *nT*_*b*_) for *nT*_*b*_ < *t* ≤ *nT*_*b*_ + Δ*T* and *v*_*i*_(*t*) = *A* for *nT*_*b*_ + Δ*T* < *t* ≤ (*n* + 1)*T*_*b*_, respectively. Then, the output signal is

(18) vo(t)=−A+2AΔT(t−nTb−RC) +(2AΔTRC+ve,n)e−(t−nTb)/RCfor  nTb<t≤nTb+ΔT,

(19) vo(t)=vo(nTb+ΔT) +{A−vo(nTb+ΔT)}(1−e−(t−nTb−ΔT)/RC)for  nTb+ΔT<t≤(n+1)Tb.

**Figure 8:**
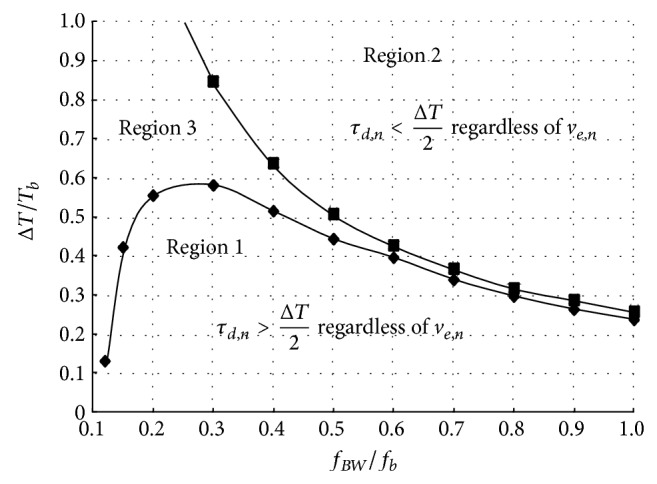
Sufficient conditions for *τ*_*d*,*n*_ > Δ*T*/2 and *τ*_*d*,*n*_ < Δ*T*/2 regardless of *v*_*e*,*n*_.

By using that *v*_*o*_((*n* + 1)*T*_*b*_) = *A* − *v*_*e*,*n*+1_, the relationship between *v*_*e*,*n*_ and *v*_*e*,*n*+1_ is obtained as

(20) $$\begin{array}{c} v_{e,n+1}=2ASr-v_{e,n}r, \end{array}$$

where *r* = *e* ^−(*T*_*b*_/RC)^ and *S* = (RC/Δ*T*)(*e* ^Δ*T*/RC^ − 1). Also, by using that *v*_*o*_(*nT*_*b*_ + (Δ*T*/2) + *τ*_*d*,*n*_) = 0, *τ*_*d*,*n*_ can be obtained as a function of *v*_*e*,*n*_. However, to solve *v*_*o*_(*nT*_*b*_ + (Δ*T*/2) + *τ*_*d*,*n*_) = 0, (18) should be used if *τ*_*d*,*n*_ < Δ*T*/2 and (19) should be used if *τ*_*d*,*n*_ > Δ*T*/2. Because *v*_*o*_(*nT*_*b*_ + Δ*T*) < 0 is equivalent to *τ*_*d*,*n*_ > Δ*T*/2 in Figure 7(a), we can say that (18) should be used if *v*_*o*_(*nT*_*b*_ + Δ*T*) > 0 and (19) should be used if *v*_*o*_(*nT*_*b*_ + Δ*T*) < 0.  Figure 8 shows the sufficient condition for *τ*_*d*,*n*_ > Δ*T*/2* regardless of v*_*e*,*n*_ as region 1 and *τ*_*d*,*n*_ < Δ*T*/2* regardless of v*_*e*,*n*_ as region 2, respectively. Region 3 is located between region 1 and region 2, in which *τ*_*d*,*n*_ can be larger or less than Δ*T*/2* depending on v*_*e*,*n*_. The regions 1, 2, and 3 of Figure 8 can be obtained by solving *v*_*o*_(*nT*_*b*_ + Δ*T*) = 0. Here, *f*_BW_ is the 3 dB bandwidth of the first order low pass system which is defined as *f*_BW_ = 1/2*π*RC. Thus, *τ*_*d*,*n*_ is obtained as

(21) $$\begin{array}{c} \tau_{d,n}=T_{b}\;\text{lo}{\text{g}}_{r}\left(\frac{A}{2AS-v_{e,n}}\right)-\frac{\Delta T}{2} \end{array}$$

from (19) in region 1 and

(22) $$\begin{array}{c} \tau_{d,n}=T_{b}\;\text{lo}{\text{g}}_{r}\left\{\frac{\left(2A/\Delta T\right)\left(\text{RC}-\tau_{d,n}\right)}{\left(2A/\Delta T\right)\text{RC}+v_{e,n}}\right\}-\frac{\Delta T}{2} \end{array}$$

from (18) in region 2, respectively. In region 3, either (21) or (22) should be appropriately chosen for *τ*_*d*,*n*_ but after both (21) and (22) are evaluated and compared with Δ*T*/2 because (21) is used if *τ*_*d*,*n*_ > Δ*T*/2 and (22) is used if *τ*_*d*,*n*_ < Δ*T*/2. Second, when the bit transition pattern is 00 as shown in Figure 7(b), the initial condition and the input signal are equal to those of Figure 3(b) so that the same relationship between *v*_*e*,*n*_ and *v*_*e*,*n*+1_ is obtained:

(23) $$\begin{array}{c} v_{e,n+1}=v_{e,n}r. \end{array}$$

Finally, when the bit transition pattern is 10 or 11 as shown in Figures 7(c) or 7(d), the same relationship between *v*_*e*,*n*_ and *v*_*e*,*n*+1_ can be obtained as (20) or (23) and *τ*_*d*,*n*_ can be obtained from (21) or (22) because Figures 7(c) and 7(d) are just vertically symmetric with Figures 7(a) and 7(b), respectively.

### 3.2. DDJ Calculation

As seen from (20) and (23), *v*_*e*,*n*_ has Markov property also when the input rise/fall time is Δ*T*. Thus, *v*_*e*,*n*_ can be generally represented as

(24) $$\begin{array}{c} v_{e,n}=2ASr\frac{\sum_{i=1}^{K}{\left(-1\right)}^{i+1}r^{a_{i,n}}}{1-r^{N}} \end{array}$$

for any data pattern with the finite pattern length of *N* by following the same steps explained in Section 2.2. Here, *K* is the number of bit transitions within the data pattern and *a*_*i*,*n*_ is an integer variable defined as the relative bit distance of the *i*th bit transition backwards from *v*_*e*,*n*_, where *a*_*i*,*n*_ ∈ {0,1,…, *N* − 1}. The only difference between (24) and (12) is the additional multiplication factor, *S*, in (24). So, *v*_*e*,max⁡_ and *v*_*e*,min⁡_ can be found among {*v*_*e*,1_, *v*_*e*,2_,…, *v*_*e*,*N*_} by comparaing only *a*_*i*,*n*_ values based on the same theorems stated in Section 2.2. Of course, if a data pattern is random and has the infinite pattern length, *v*_*e*,min⁡_ = 0 and *v*_*e*,max⁡_ = 2*ASr*. For any data pattern, *τ*_*d*,max⁡_ and *τ*_*d*,min⁡_ can be obtained from either (21) or (22) depending on *f*_BW_/*f*_*b*_ and Δ*T*/*T*_*b*_. If *f*_BW_/*f*_*b*_ and Δ*T*/*T*_*b*_ are in region 1 of Figure 8,

(25) $$\begin{array}{c} \tau_{d,\max}=T_{b}\;\text{lo}{\text{g}}_{r}\left(\frac{A}{2AS-v_{e,\min}}\right)-\frac{\Delta T}{2}\\ \tau_{d,\min}=T_{b}\;\text{lo}{\text{g}}_{r}\left(\frac{A}{2AS-v_{e,\max}}\right)-\frac{\Delta T}{2} \end{array}$$

from (21) and if *f*_BW_/*f*_*b*_ and Δ*T*/*T*_*b*_ are in region 2,

(26) $$\begin{array}{c} \tau_{d,\max}=T_{b}\;\text{lo}{\text{g}}_{r}\left\{\frac{\left(2A/\Delta T\right)\left(\text{RC}-\tau_{d,\max}\right)}{\left(2A/\Delta T\right)\text{RC}+v_{e,\min}}\right\}-\frac{\Delta T}{2}\\ \tau_{d,\min}=T_{b}\;\text{lo}{\text{g}}_{r}\left\{\frac{\left(2A/\Delta T\right)\left(\text{RC}-\tau_{d,\min}\right)}{\left(2A/\Delta T\right)\text{RC}+v_{e,\max}}\right\}-\frac{\Delta T}{2} \end{array}$$

from (22). Otherwise, if *f*_BW_/*f*_*b*_ and Δ*T*/*T*_*b*_ are in region 3, the appropriate equations should be chosen from (25) and (26) for calculation of *τ*_*d*,max⁡_ and *τ*_*d*,min⁡_ by comparing the calculated values of *τ*_*d*,max⁡_ and *τ*_*d*,min⁡_ with Δ*T*/2. Consequently, the DDJ is derived as

(27) $$\begin{array}{c} \text{DDJ}=\tau_{d,\max}-\tau_{d,\min}=T_{b}\;\text{lo}{\text{g}}_{r}\left(\frac{2AS-v_{e,\max}}{2AS-v_{e,\min}}\right) \end{array}$$

in region 1 and the DDJ is calculated by using (26) in region 2. Although (27) and (14) look a bit different, the DDJ equation of (27) equals the DDJ equation of (14) since *v*_*e*,min⁡_ and *v*_*e*,max⁡_ are linearly proportional to the multiplication factor, *S*, as shown in (24) and thus *S* is cancelled out from (27). This means that the DDJ value does not depend on Δ*T* in region 1 and equals the DDJ value when Δ*T* = 0. On the other hand, the DDJ value in region 2 is slightly larger than the DDJ value when Δ*T* = 0. However, the difference is quite small and acceptable because the system bandwidth is relatively large compared to the bit rate in region 2 as shown in Figure 8 and so the DDJ value is very small in itself.

## 4. Simulation Results

**Figure 9:**
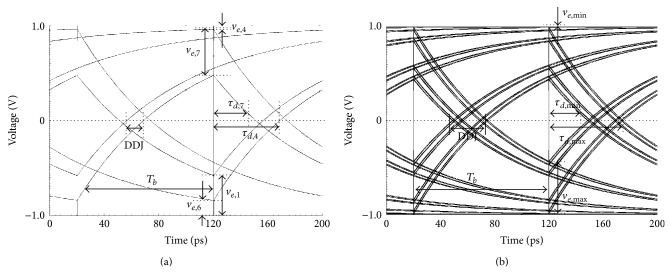
Eye diagrams of the simulated input and output waveforms when the input data pattern is (a) PRBS3 and (b) random. The input rise/fall time is zero. *T*_*b*_ = 100* *ps and *f*_BW_=2 GHz.

Figure 9 shows the eye diagrams of the simulated input and output waveforms when the data pattern is (a) PRBS3 and (b) random, respectively. The input rise/fall time is zero, the bit rate is 10 Gb/s, and the system bandwidth is 2 GHz. All the waveforms were obtained by running Cadence Spectre. In Figure 9(a), among {*v*_*e*,1_, *v*_*e*,4_, *v*_*e*,6_, *v*_*e*,7_}, *v*_*e*,max⁡_ = *v*_*e*,7_ and *v*_*e*,min⁡_ = *v*_*e*,4_, as discussed in Section 2.2. So, *τ*_*d*,max⁡_ = *τ*_*d*,4_, *τ*_*d*,min⁡_ = *τ*_*d*,7_, and DDJ = *τ*_*d*,4_ − *τ*_*d*,7_. In Figure 9(b), *v*_*e*,min⁡_ = 0 and *v*_*e*,max⁡_ = 2*Ar*, and *τ*_*d*,max⁡_ and *τ*_*d*,min⁡_ correspond to *v*_*e*,min⁡_ and *v*_*e*,max⁡_, respectively. All the simulated *v*_*e*,max⁡_, *v*_*e*,min⁡_, *τ*_*d*,max⁡_, *τ*_*d*,min⁡_, and DDJ for various data patterns agree exactly with the calculated values in Table 2.

**Figure 10:**
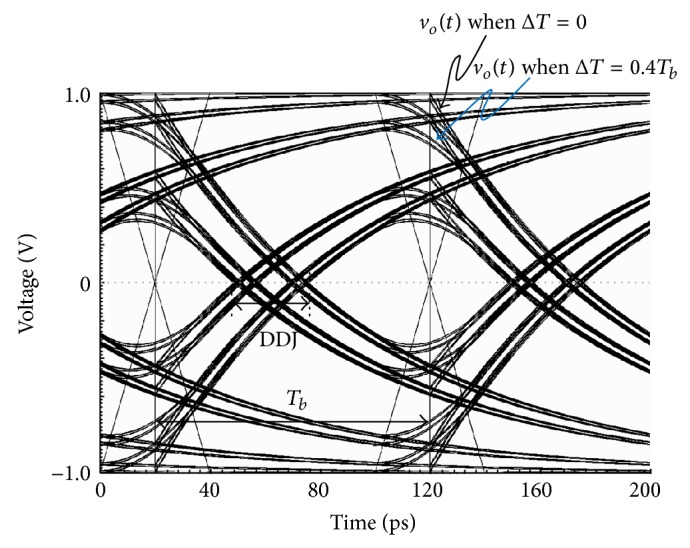
Comparison of eye diagrams of the output waveforms when Δ*T* = 0 and Δ*T* = 0.4*T*_*b*_. *T*_*b*_ = 100 ps and *f*_BW_ = 2 GHz.

Figure 10 compares the eye diagrams of the simulated output waveforms for Δ*T* = 0 and Δ*T* = 0.4*T*_*b*_. The data pattern is random, the bit rate is 10 Gb/s, and the system bandwidth is 2 GHz. The input signal, of which rise/fall time is modeled by Δ*T* as first order approximation, is applied to the first order low pass system. As shown in Figure 10, two output waveforms slightly differ from each other only around the transition edges of the input signals; however, they exactly coincide with each other around the transition edges of the output signals. Thus, the simulated DDJ when Δ*T* = 0.4*T*_*b*_ equals the simulated DDJ when Δ*T* = 0. This coincidence between Δ*T* = 0 and Δ*T* = 0.4*T*_*b*_ is due to the fact that *f*_BW_/*f*_*b*_ and Δ*T*/*T*_*b*_ are in region 1.

**Figure 11:**
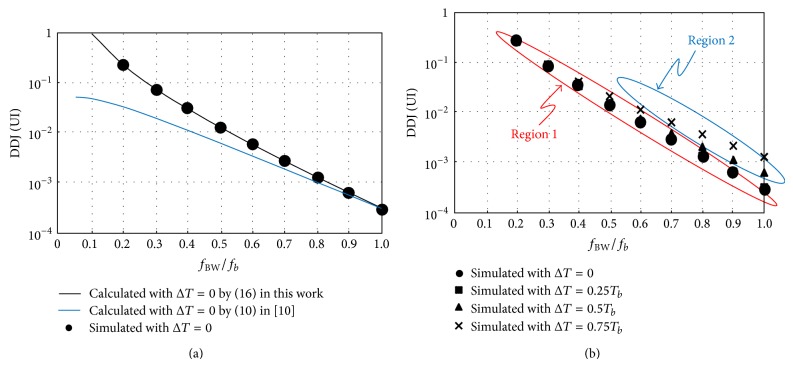
(a) Comparison of the DDJ values calculated by (16) in this work and by (10) in [10] and (b) the simulated DDJ values versus various input rise/fall times.

Figure 11(a) shows the comparison of the simulated DDJ with the calculated DDJ by (16) in this work and the calculated DDJ by (10) in [10]. The calculated DDJ in this work better estimates the simulated DDJ. As the system bandwidth, *f*_BW_, decreases, the calculated DDJ by (10) in [10] underestimates the simulated DDJ because (10) in [10] was derived by using only two preceding bits as discussed in Appendix C.  Figure 11(b) shows the simulated DDJ for various input rise/fall times. Δ*T* varies from 0 to 0.75*T*_*b*_. If *f*_BW_/*f*_*b*_ and Δ*T*/*T*_*b*_ are in region 1, the simulated DDJ does not depend on Δ*T*; however, in region 2, the simulated DDJ starts to deviate as Δ*T* increases. Even in this case, the deviation of the simulated DDJ is smaller than 0.008 UI because the absolute DDJ value is very small in itself, that is, less than 0.01 UI, in region 2. The system bandwidth, *f*_BW_, is relatively large compared to the bit rate, *f*_*b*_, in region 2 as shown in Figure 8.

Additionally, the simulated DDJ values when the low pass system has the additional second pole, *f*_*p*2_, are summarized in Table 3. As shown in the table, the calculated DDJ values by the derived equations agree well with the simulated results with the accuracy of less than 0.021 UI when the second pole is larger than 5 times of the first pole, that is, 10 GHz. However, if the second pole approaches the first pole, the system bandwidth now decreases less than the first pole so that the simulated results start to deviate from the calculated DDJ values.

## 5. Conclusion

The closed-form equation of DDJ in a first order low pass system has been derived. If the bit rate, the system bandwidth, the input rise/fall time, and the number of maximum consecutive identical bits are given, the DDJ can be calculated exactly in region 1 and accurately in regions 2 and 3. The simulated DDJ agrees well with the calculated DDJ. Because the DDJ in the transmission line may apply to the interconnect channels such as off-chip PCB traces, off-chip cables, and on-chip interconnect lines and the DDJ in the first order low pass system may apply to the transceiver circuit building blocks such as drivers, buffers, amplifiers, and limiters, the derived equation can be generally used for a high speed serial interface when we design system architecture, link budget, and each circuit building block which can be modeled as a first order low pass system.

## Figures and Tables

**Table 1:** Definition of the variables.

Variable	Definition
*f*_BW_	3 dB bandwidth of the first order low pass system
*T*_*b*_	Bit duration
*f*_*b*_	Bit rate, 1/T_b_
Δ*T*	Rise/fall time of the input signal
*v*_*e*,*n*_	Voltage difference between *v*_*i*_(*t*) and *v*_*o*_(t) at *t* = nT_b_
τ_*d*,*n*_	Time difference between the threshold crossing times of *v*_*i*_(*t*) and *v*_*o*_(*t*) for *nT*_*b*_ < *t* < (*n* + 1)*T*_*b*_
*a*_*i*,*n*_	Relative bit distance of the *i*th bit transition backwards from *v*_*e*,*n*_
*N*	Pattern length
*K*	Number of bit transitions

**Table 2:** Calculated *v*_*e*,max⁡_, *v*_*e*,min⁡_, *τ*_*d*,max⁡_, *τ*_*d*,min⁡_, and DDJ values for various data patterns. The input rise/fall time is zero. *T*_*b*_ = 100 ps and *f*_BW_ = 2 GHz.

Data pattern	PRBS3	PRBS4	PRBS5	Random
*v*_*e*,max⁡_ (V)	0.52	0.56	0.57	0.57
*v*_*e*,min⁡_ (V)	0.04	0.01	0.00	0.00
*τ*_*d*,max⁡_ (ps)	53.70	54.75	55.02	55.16
*τ*_*d*,min⁡_ (ps)	30.99	29.23	28.67	28.51
DDJ (ps)	22.71	25.52	26.35	26.65

**Table 3:** Simulated DDJ values when the low pass system has the additional second pole, *f*_*p*2_. The input rise/fall time is zero. *T*_*b*_ = 100 ps and *f*_*p*1_ = 2 GHz.

Second pole	Data pattern			
	PRBS3	PRBS4	PRBS5	Random
No second pole	22.71	25.52	26.35	26.65
*f*_*p*2_ = 20 GHz	22.81	25.68	26.52	26.83
*f*_*p*2_ = 10 GHz	24.35	27.48	28.41	28.75
*f*_*p*2_ = 5 GHz	32.23	36.86	38.48	38.81

## Appendices

### A. Proofs of Theorems 1, 2, and 3

Proof of Theorem 1. Subtracting $v_{e,m}$ from $v_{e,n}$ leads to

(A.1)

because $r^{a_{i,n}}-r^{a_{i+1,n}}>0$. By using the fact that $r^{a_{1,n}}-r^{a_{2,n}}-r^{a_{1,m}}$ is minimum when $a_{2,n}=a_{1,n}+1$ and $a_{1,m}=a_{1,n}+1$,

(A.2)

because r is smaller than 0.5 as far as the system bandwidth, $f_{\text{BW}}$, is larger than 11% of the bit rate, $f_{b}=1/T_{b}$, which is typical of the high speed serial interface systems [8].

Proof of Theorem 2. Subtracting $v_{e,n}$ from $v_{e,m}$ leads to

(A.3)

because $r^{a_{i,n}}-r^{a_{i+1,n}}>0$. By using the fact that $-r^{a_{2k,m}}+r^{a_{2k,n}}-r^{a_{2k+1,n}}$ is minimum at $a_{2k,m}=a_{2k,n}+1$ and $a_{2k+1,n}=a_{2k,n}+1$,

(A.4)

because r is smaller than 0.5.

Proof of Theorem 3. Subtracting $v_{e,m}$ from $v_{e,n}$ leads to

(A.5)

because $r^{a_{i,n}}-r^{a_{i+1,n}}>0$. By using the fact that $r^{a_{2k+1,n}}-r^{a_{2k+2,n}}-r^{a_{2k+1,m}}$ is minimum at $a_{2k+2,n}=a_{2k+1,n}+1$ and $a_{2k+1,m}=a_{2k+1,n}+1$,

(A.6)

because r is smaller than 0.5.

### B. Closed-Form DDJ Equations of PRBS4 and PRBS5

Following the same steps explained in Section 2.2, the DDJ can be derived for other PRBS data. For PRBS4

(B.1)

and for PRBS5

(B.2)

### C. Comments on the DDJ Equation of (10) in [10]

In [10], the closed-form DDJ equation of (10) has been calculated for the first order low pass system by considering only two preceding bits, $a_{-1}$ and $a_{-2}$, as follows:

(C.1)

where $\tau =1/2\pi f_{\text{BW}}$ and $\alpha =e^{-2\pi f_{\text{BW}}T_{b}}$. However, as the bit rate increases relatively to the system bandwidth, α increases and the impact of additional bits such as $a_{-3}$ and $a_{-4}$ should be considered as shown in Figure 3 of [10]. Consequently, the calculated DDJ by (10) in [10], which is rewritten in (C.1), underestimates the simulated DDJ as shown in Figure 11(a).

## References

[1] Lee W.-Y., Kim L.-S. (2012). A 5.4-Gb/s clock and data recovery circuit using seamless loop transition scheme with minimal phase noise degradation. *IEEE Transactions on Circuits and Systems I*.

[2] Byun S., Son C. H., Hwang J., Min B.-H., Park M.-Y., Yu H.-K. (2013). 1–5.6 Gb/s CMOS clock and data recovery IC with a static phase offset compensated linear phase detector. *IET Circuits, Devices & Systems*.

[3] Liu H., Wang Y., Xu C., Chen X., Lin L., Yu Y., Wang W., Majumder A., Chui G., Brown D., Fang A. (2014). A 5-Gb/s serial-link redriver with adaptive equalizer and transmitter swing enhancement. *IEEE Transactions on Circuits and Systems I*.

[4] Chen M.-S., Shih Y.-N., Lin C.-L., Hung H.-W., Lee J. (2012). A fully-integrated 40-Gb/s transceiver in 65-nm CMOS technology. *IEEE Journal of Solid-State Circuits*.

[5] (2004). Fibre channel-methodologies for jitter and signal quality specification (MJSQ). *Technical Report Review*.

[6] Maxim Application Note 1916 An introduction to jitter in communications systems. http://www.maximintegrated.com/app-notes/index.mvp/id/1916.

[7] Yu W., Shi R., Cheng C.-K. (2009). Accurate eye diagram prediction based on step response and its application to low-power equalizer design. *IEICE Transactions on Electronics*.

[8] Shi R., Yu W., Zhu Y., Cheng C.-K., Kuh E. S. Efficient and accurate eye diagram prediction for high speed signaling.

[9] Kim D., Kim H., Eo Y. (2012). Analytical eye-diagram determination for the efficient and accurate signal integrity verification of single interconnect lines. *IEEE Transactions on Computer-Aided Design of Integrated Circuits and Systems*.

[10] Buckwalter J., Analui B., Hajimiri A. (2004). Predicting data-dependent jitter. *IEEE Transactions on Circuits and Systems II: Express Briefs*.

[11] Analui B., Buckwalter J. F., Hajimiri A. (2005). Data-dependent jitter in serial communications. *IEEE Transactions on Microwave Theory and Techniques*.

[12] Hong D., Cheng K. T. An accurate jitter estimation technique for efficient high speed I/O testing.

[13] Buckwalter J. F., Hajimiri A. (2006). Analysis and equalization of data-dependent jitter. *IEEE Journal of Solid-State Circuits*.

[14] Xia T., Mu D. (2010). High speed interconnect data dependent jitter analysis. *Microelectronics Journal*.

[15] Papoulis A., Pillai S. U. (2002). *Probability, Random Variables and Stochastic Processes*.

[16] Miller S., Childers D. (2012). *Probability and Random Process*.

